# Acute kidney injury and tacrolimus toxicity in a kidney transplant recipient treated with nirmaltrevir/ritonavir: a case report

**DOI:** 10.1186/s13256-024-04990-6

**Published:** 2024-12-20

**Authors:** Jack Rycen, Julia Jefferis, David Mudge

**Affiliations:** 1 Department of Kidney and Transplant Services, Princess Alexandra Hospital, Brisbane, Australia; 2 Faculty of Medicine, The University of Queensland, Brisbane, Australia

**Keywords:** Paxlovid, COVID-19, Kidney transplant, AKI, Case report

## Abstract

**Background:**

Kidney transplant recipients with severe acute respiratory syndrome-coronavirus-2 infection have an increased risk of severe disease and mortality. Nirmaltrevir/ritonavir (Paxlovid) is an effective oral disease-modifying therapy that has been shown to reduce risk of progression to severe disease in high-risk, nonhospitalized adults. However, owing to the potential for serious drug–drug interactions owing to ritonavir-induced inhibition of the CYP3A enzyme, this drug is not suitable option for transplant recipients with mild-moderate severe acute respiratory syndrome-coronavirus-2 infection.

**Case presentation:**

A 57-year-old Caucasian man presented to the emergency department with 48 hours of nausea, vomiting, headaches, and lethargy. At 5 days earlier, he was diagnosed with a mild severe acute respiratory syndrome-coronavirus-2 infection by his general practitioner, who commenced treatment with Paxlovid at 300 mg/100 mg twice daily. Past medical history included kidney transplantation in 2018 for end-stage kidney secondary to hypertensive nephrosclerosis, managed with prednisone, tacrolimus, and mycophenolate. Vaccination status was up-to-date and prophylactic tixagevimab/cilgavimab (Evusheld) had been given > 6 months prior owing to lack of seroconversion. Examination showed a blood pressure of 176/94 mmHg and normal respiratory parameters. Investigations demonstrated a serum creatinine of 213 µmol/L (baseline 130 µmol/L) and tacrolimus trough level of 118 µg/L (baseline 6.9–8.7 µg/L). Treatment included intravenous rehydration, Evusheld and tacrolimus were withheld for 7 days, with recommencement guided by regular therapeutic drug monitoring.

**Conclusion:**

This acute kidney injury was attributed to tacrolimus toxicity resulting from a drug–drug interaction with Paxlovid. While transplant recipients have an increased risk of severe disease, current Australian guidelines recommend against Paxlovid use in adults taking medications that are heavily dependent on CYP3A4 for clearance, including calcineurin and mammalian target of rapamycin inhibitors.

## Introduction

Compared with the general population, kidney transplant recipients with severe acute respiratory syndrome-coronavirus-2 (SARS-CoV-2) infection have an increased risk of severe disease and mortality [1]. In Australia, the Therapeutic Goods Administration has approved two oral disease-modifying therapies for patients who do not require supplemental oxygen and have one or more risk factors for disease progression within 5 days of symptom onset: nirmatrelvir/ritonavir (Paxlovid) and molunpiravir (Lageviro). Paxlovid is a combination of nirmatrelvir, a peptidomimetic inhibitor of the coronavirus 3C-like (3CL) protease, and ritonavir, an antiretroviral drug that inhibits the CYP3A4 isozyme to maintain plasma nirmatrelvir trough concentrations at effective levels. While clinical trial data has demonstrated a significant reduction in hospitalization and mortality in high-risk individuals treated with Paxlovid, its use has been limited by the potential for serious adverse drug–drug interactions with co-medications that are dependent on the CYP3A4 enzyme for metabolism [2, 3]. We present the case of a kidney transplant patient who developed tacrolimus toxicity with an acute kidney injury (AKI) following a drug–drug interaction with Paxlovid.

## Case presentation

We present the case of a 57-year-old Caucasian man with end-stage kidney disease presumed secondary to hypertensive nephrosclerosis who underwent a successful deceased donor (donation after brain death) kidney transplant in June 2018. Tissue typing revealed no human leukocyte antigen (HLA) mismatches or donor-specific antibodies. Early post-transplant complications included *Klebsiella pneumoniae* transplant and native kidney pyelonephritis with accompanying bloodstream infection and new-onset diabetes after transplantation (NODAT). His immunosuppressive regimen consisted of 2 mg of immediate release tacrolimus (Prograf) twice daily, 720 mg of mycophenolate sodium (Myfortic) twice daily, and 7 mg of prednisone once daily. His baseline serum creatinine ranged from 118 to 140 (median: 130) µmol/L and tacrolimus trough level was maintained between 6.9 and 8.7 µg/L, measured using high performance liquid chromatography mass spectrometry (HPC-MS).

His past medical history included complicated Crohn’s disease resulting in a colectomy and ileostomy in 2012, hypertension, well-controlled asthma and obesity. Other regular medications included 100 mg of aspirin daily, 50 mg of metoprolol twice daily, 20 mg of esomeprazole daily, 30 mg of gliclazide modified release (MR) daily, 1000 mg of metformin MR daily, 1 mg of semaglutide weekly, 80/400 mg of trimethoprim-sulfamethoxazole daily (continued indefinitely as per local transplant unit policy) and 840 mg of sodium bicarbonate twice daily.

He presented to a tertiary emergency department in the evening with nausea, vomiting, headache, and generalized weakness over the preceding 48 hours. There was no history of abdominal pain, altered bowel habits, dysuria, fevers or recent travel. He had been adherent to all of his regular medications as listed above. At 5 days prior to the current presentation, he had developed rhinorrhea and a nonproductive cough and was diagnosed with mild coronavirus disease 2019 (COVID-19) using rapid antigen testing. His COVID-19 vaccinations included two doses of AstraZeneca Vaxzevria (April and July 2021) and a single dose of the Pfizer Comirnaty booster (January 2022). He had previously received a single dose of tixagevimab/cilgavimab (Evusheld) for pre-exposure prophylaxis owing to lack of seroconversion [anti-S and anti-N immunoglobulin G (IgG) negative in July 2022]. He attended his local general practitioner who prescribed a 5-day course of nirmatrelvir/ritonavir (Paxlovid) at 300 mg/100 mg twice daily. The patient had completed 4 days of treatment before he was unable to tolerate oral intake owing to the onset of the abovementioned symptoms.

Clinical examination was remarkable for hypertension with a blood pressure of 176/94 mmHg. There was no respiratory involvement, fevers, or hypoxia (temperature of 36.1 °C and oxygen saturation of 99% on ambient air). His weight was 104.4 kg [body mass index (BMI) 32 kg/m^2^]. The abdomen was soft and nondistended with no focal tenderness over the transplanted kidney. Tremors were reported by the patient but not observed and there was no focal neurology. Laboratory investigations showed a serum creatinine level of 213 µmol/L, potassium of 5.3 mmol/L, and corrected calcium of 2.78 mmol/L (range 2.10–2.60 mmol/L). Blood glucose level was 13 mmol/L and liver tests were normal. Urinalysis was negative for leukocytes and erythrocytes and no bacteria was cultured. Blood cultures were also negative. A tacrolimus trough level was requested for the following morning.

Initial treatment received in the emergency department (ED) included 1000 mL of intravenous sodium chloride 0.9%, a single dose of 100 mg of intravenous hydrocortisone, 1000 mg of intravenous paracetamol, and 4 mg of intravenous ondansetron. The patient was then able to tolerate his regular immunosuppressive medications that night. After consultation with the local infectious diseases team, a second dose of 300/300 mg of intra-muscular Evusheld was administered as a single dose.

**Fig. 1:**
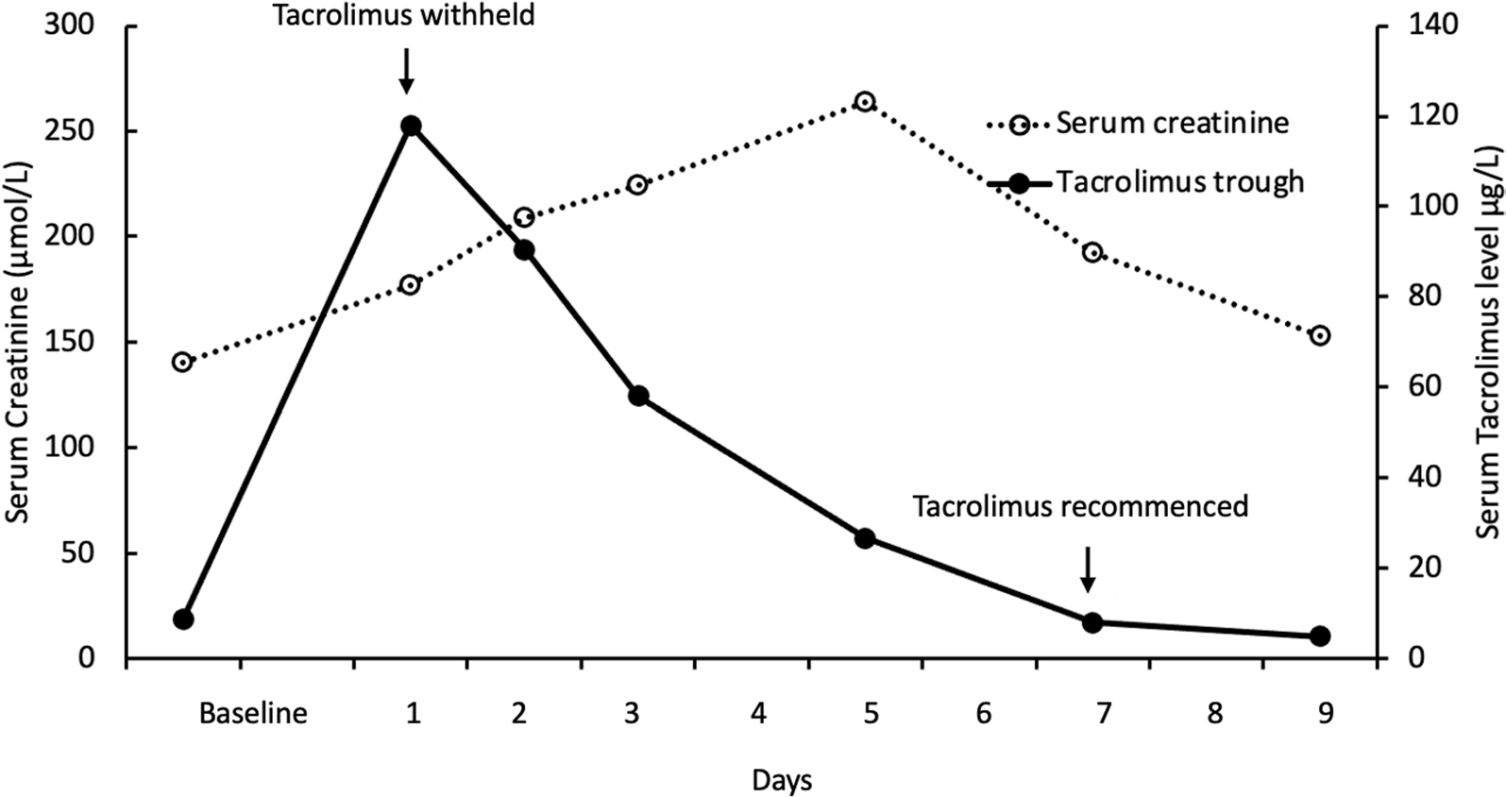
Timeline of serum creatinine and trough tacrolimus levels after Paxlovid therapy

Repeat biochemistry on day 1 of admission demonstrated an improvement in serum creatinine level (177 µmol/L) and corrected calcium level (2.67 mmol/L), likely confirming an element of volume depletion from profuse vomiting. The patient reported a marked improvement in his symptoms and was able to tolerate his normal diet. His blood pressure improved to 136/78 mmHg. The patient was discharged with a phone review the following day. Later that afternoon, the tacrolimus trough level returned elevated at 118 µg/L (Fig. 1). The patient was instructed to withhold his tacrolimus and remain well hydrated. He was managed as an outpatient with serial phone reviews every 48 hours along with serum biochemistry and therapeutic drug monitoring. During the follow up period, his creatinine level peaked at 264 µmol/L on day 5 and he had no deterioration to suggest progression to moderate or severe COVID-19. In total, tacrolimus was withheld for 6 days before being recommenced at 100% of his regular dose (2 mg twice daily) once the trough level was back in target range.

## Discussion

Paxlovid remains an important part of the pharmacotherapeutic armamentarium for high-risk, nonhospitalized adults with mild-moderate COVID-19, especially given that emerging evidence suggests that alternative therapies, including Lageviro and Evusheld, are no longer effective at reducing important COVID-19-related outcomes in the face of newer viral variants [4, 5]. Kidney transplant patients with mild-moderate COVID-19 present a challenge to manage given the increased risk of progression to severe disease and associated mortality, and a lack of suitable oral therapies that can be delivered in a community-based setting. Our case illustrates the interaction between ritonavir and tacrolimus, a calcineurin inhibitor and integral part of a standard three-drug immunosuppressive regimen to prevent allograft rejection in kidney transplantation.

Tacrolimus (formerly known as FK506) is a macrolide antibiotic originally derived from *Streptomyces tsukubensis.* Through binding to immunophilin FKBP-12, it inhibits calcineurin and other important signaling pathways required for interleukin 2 (IL-2) production and T cell activation. It is a substrate of the intestinal counter-transporter, P-glycoprotein, and is metabolized extensively in the liver by the CYP3A subfamily, primarily CYP3A4 [6]. Several factors have been shown to affect plasma tacrolimus concentrations, including patient demographics (age, sex, and ethnicity), liver and kidney function, polymorphisms in the CYP3A isozymes, and coadministered food and medications. Multiple medications have the potential to increase plasma tacrolimus concentrations to supratherapeutic and toxic levels, largely through inhibition of P-glycoprotein and/or the CYP3A isozymes. Potent CYP3A inhibitors include macrolide antibiotics, non-dihydropyridine calcium channel blockers, azole antifungals, and antiretrovirals, such as ritonavir [7]. The latter has been shown to have a rapid inhibitory effect that can increase plasma concentrations of CYP3A-dependent medications between 1.8- and 20-fold [8]. Pharmacokinetic studies have also shown that this effect may be prolonged with 70–90% of enzyme recovery expected between 2 and 5 days [9].

In our patient, the plasma trough tacrolimus concentration reached a peak of 118 µg/L after receiving 4 days of Paxlovid; this was a 13-fold increase from average baseline levels and took a total of 7 days to return to target range. Alternative causes, such as severe diarrhea, anemia or low hematocrit, inadvertent overdose, or other medications known to interact with tacrolimus, were sought and not identified. Notably, he had received a full-strength dose of Paxlovid (300 mg/100 mg every 12 hours), which is recommended for patients with an estimated glomerular filtration rate (eGFR) > 60 mL/min, despite his baseline eGFR being 50 mL/min. Clinical manifestations of acute tacrolimus toxicity were evident and included KDIGO Stage 2 AKI, nausea and vomiting, headache, hypertension, electrolyte disturbances, and reported fine tremors. Importantly, his kidney function returned to near baseline after 9 days from presentation with supportive care and temporary cessation of tacrolimus alone.

Interestingly, the use of CYP3A4 inducers, including phenytoin and rifampin, has been described in patients with severe manifestations of tacrolimus toxicity. In a case series by Jantz *et al*., four solid organ transplant recipients with supratherapeutic plasma tacrolimus concentrations (> 30 ng/L) and AKI (serum creatinine 67 to 630% from baseline) with severe hyperkalemia (6.1–6.7 mmol/L) were treated with an average of 3 days of oral phenytoin at 400 mg per day to promote tacrolimus metabolism. In all four patients, plasma tacrolimus concentration rapidly decreased to < 15 ng/L after 3 days and kidney function returned to baseline between 3 and 10 days in three patients. No clinically significant adverse effects were reported [10].

While risk mitigation strategies (that is, dose reduction of calcineurin inhibitors guided by therapeutic drug monitoring) have been proposed outside of clinical guidelines to allow for Paxlovid use in kidney transplant recipients, we would advocate against this practice owing to the potential for harmful drug-drug interactions and logistic difficulties associated with regular therapeutic drug monitoring in community-based adults, particularly for those who live outside of major metropolitan areas [11] 12. In addition, there is a lack of high-quality evidence to support Paxlovid use in transplant recipients given adults who require CYP3A4-dependent medications (that is, calcineurin inhibitors) were excluded from the EPIC-HR trial, which notably demonstrated a substantial 89% reduction in progression to severe disease in unvaccinated adults when B.1.617.2 (Delta) was the prevalent circulating variant [2]. This patient population was also excluded from a large US population-based cohort study, which demonstrated a 44% reduction in hospitalization and death among nonhospitalized adults treated with Paxlovid during the B.1.1.529 (Omicron) pandemic, where over 90% of the study population had received three or more vaccines [13].

## Conclusion

This case highlights the potential safety issues associated with the coadministration of Paxlovid and calcineurin inhibitors. Furthermore, prescribers have a responsibility to ensure that no interactions exist among high-risk medications and should utilize the assistance of the transplant pharmacist as well as online-based decision-making tools associated with clinical guidelines.

## Data Availability

Data sharing is not applicable to this article as no datasets were generated or analysed during the current study.
